# Diagnosis values of Dectin-1 and IL-17 levels in plasma for invasive pulmonary aspergillosis in bronchiectasis

**DOI:** 10.3389/fcimb.2022.1018499

**Published:** 2022-10-11

**Authors:** Qian He, Min Li, Jiaqi Cao, Ming Zhang, Chunlai Feng

**Affiliations:** Department of Respiratory and Critical Care Medicine, Third Affiliated Hospital of Soochow University, Changzhou, China

**Keywords:** invasive pulmonary aspergillosis, bronchiectasis, Dectin-1, IL-17, diagnosis values

## Abstract

**Background:**

Among immunocompetent patients, patients with bronchiectasis are considered to be a high-risk group for invasive pulmonary aspergillosis (IPA). Early diagnosis and treatment can improve the prognosis of patients.

**Objectives:**

We aimed to investigate the diagnostic value of Dectin-1 and IL-17 for diagnosing IPA with bronchiectasis.

**Methods:**

We retrospectively collected data on patients with bronchiectasis who had been hospitalized in the Third Affiliated Hospital of Soochow University between September 2018 to December 2021. Dectin-1, IL-17 and GM were measured by enzyme-linked immunosorbent assays.

**Results:**

A total of 129 patients were analyzed in the study, of whom 33 had proven or probable IPA with bronchiectasis. The remaining 96 patients served as controls. The plasma Dectin-1 and IL-17 levels in the IPA group were significantly higher than that in the control group (P=0.005; P<0.001). The plasma GM, BALF GM, plasma Dectin-1 and IL-17 assays had sensitivities of 39.4%, 62.5%, 69.7% and 78.8%, respectively, and specificities of 89.2%, 91.5%, 72.9% and 71.9%, respectively. The sensitivity of Dectin-1 and IL-17 in plasma was higher than that in plasma and BALF GM. while the specificity is lower than that of plasma and BALF GM. The diagnostic sensitivity and specificity of plasma GM combined with IL-17 for IPA in bronchiectasis were greater than 80%. The combination of plasma GM and IL-17 can improve the sensitivity of the GM test, but does not reduce the diagnostic specificity. The plasma Dectin-1 and IL-17 showed positive linear correlations with the bronchiectasis severity Index (BSI) score in linear regression.

**Conclusions:**

Plasma Dectin-1 and IL-17 levels were significantly higher in bronchiectasis patients with IPA. The sensitivity of Dectin-1 and IL-17 was superior to that of GM for the diagnosis of IPA in patients with bronchiectasis. The combination of GM and IL-17 in plasma is helpful for the diagnosis of IPA in bronchiectasis patients who cannot tolerate invasive procedures.

## Introduction

Invasive pulmonary aspergillosis(IPA) is an infectious disease with high mortality caused by *Aspergillus spp* (Gold et al., 2022). The Cohort of Asian and Matched European Bronchiectasis (CAMEB) study found that fungi, especially *Aspergillus*, play an important role in bronchiectasis (Mac Aogáin et al., 2018). Due to airway distortion, immune dysregulation and persistent *Aspergillus* colonization exist, patients with bronchiectasis are at high risk of developing IPA (Xu et al., 2022). Non-neutropenic IPA represents a rare but important clinical phenotype. It is particularly challenging to detect IPA in non-neutropenic patients, such as IPA observed in bronchiectasis, who go undiagnosed for long periods as the disease progresses (Tiew et al., 2021; Yang et al., 2021). At present, In some studies of non-neutropenic IPA patients in China, more than 25% of the patients had bronchiectasis (Zhou et al., 2017; Wu et al., 2021). Therefore, in clinical practice, for patients with bronchiectasis who are highly suspected of IPA, multiple laboratory examination methods should be used to assist the diagnosis as much as possible.

Galactomannan (GM) is a cell wall polysaccharide of *Aspergillus* species with diagnostic importance. According to the Infectious Diseases Society of America guidelines (IDSA), the GM test in plasma or bronchoalveolar lavage fluid is considered a mycological criterion in IPA (Patterson et al., 2016). However, the GM test has limited diagnostic value in non-neutropenic IPA patients, including those with bronchiectasis (Dai et al., 2021). Dectin-1 floats in the membrane as a monomer but binds to beta-glucans as a dimer. The 176-amino-acid-long (20-kDa) extracellular C-terminal beta-glucan binding domain is often manipulated alone as soluble Dectin-1. It recognizes beta-1,3-glucan on fungal cell walls and plays a crucial role in early host defence in the respiratory tract (Kalia et al., 2021). A study has shown that dectin-1 knockout mice are more susceptible to *Aspergillus* infection, because of insufficient neutrophil recruitment in the lungs (Werner et al., 2009). Several studies have demonstrated a close association between Dectin-1 polymorphisms and susceptibility to aspergillosis (Zhou et al., 2019; Kalkanci et al., 2020). Therefore, Dectin-1 is recognized as a biomarker and target for the diagnosis and treatment of IPA. In a mouse model of *Aspergillus fumigatus* infection, Dectin-1-mediated signals can enable Th17 differentiation and increase the production of IL-17. IL-17 has been highlighted as a key component of fungal immunity. IL-17 can induce the production of antimicrobial peptides and chemokines, and lead to the recruitment of neutrophils (Puerta-Arias et al., 2020). In a study of people with severe asthma with fungal sensitization (*Aspergillus fumigatus*), it was found that BAL fungal presence by qPCR correlated with plasma IL-17 (Sullivan et al., 2020). Thus, in this study, we evaluated the diagnostic value of plasma Dectin-1 and IL-17 levels in bronchiectasis patients with IPA.

## Materials and methods

### Patients

Patients admitted to the Department of Respiratory and Critical Care Medicine at the Third Affiliated Hospital of Soochow University (Changzhou First People’s Hospital) from September 2018 to December 2021 and diagnosed with IPA with bronchiectasis and non-IPA bronchiectasis patients hospitalized were enrolled in this study. The study was approved by the Institutional Review Board of Changzhou first people’s Hospital (No.2021-009)

The inclusion criteria for the IPA with bronchiectasis were: 1. Diagnosis of bronchiectasis. 2. “proven” and “probable” invasive pulmonary aspergillosis cases that meet the diagnostic criteria of the European Organization for Research and Treatment of Cancer and the Mycoses Study Group (EORTC/MSG) (Donnelly et al., 2020). The exclusion criteria were as follows: A previous hematopoietic stem-cell or solid organ transplant or neutropenia; The non-IPA bronchiectasis patients present with bronchiectasis or concomitant infection on CT, but no relevant evidence of *Aspergillus* infection.

### Collect plasma samples and bronchoalveolar lavage fluid to detect GM, Dectin-1 and IL-17

plasma samples were collected from each subject before treatment initiation. Bronchoalveolar lavage was performed on the intrapulmonary area according to the results of the chest CT examination to obtain bronchoalveolar lavage fluid. The plasma and BALF samples were assayed for *Aspergillus* GM antigen by ELISA (Platelia *Aspergillus* Kit, Bio-Rad Laboratories). ELISA also was used to detect plasma Dectin-1 (Human Dectin-1 ELISA Kit, Thermo Fisher) and IL-17 (Human IL-17 ELISA Kit, Abcam) levels following the standard methods.

### Statistical analysis

Continuous variables were expressed as the mean standard deviation. Categorical variables were expressed as proportions. For count data and categorical variables, the chi-squared test or Fisher s exact test was used to compare groups, and for continuous variables, Student s t-tests or the Mann-Whitney U-test were used to compare groups, depending on whether or not the data were normally distributed. Receiver-operating characteristic (ROC) curve analysis was used to determine the optimal cut-off value for Dectin-1 and IL-17 for diagnosing IPA.Correlation analysis using Spearman’s correlation. All data were analyzed using SPSS version 23.0 (IBM Corp, Armonk, NY, USA). A P value<0.05 was taken to indicate statistical significance.

## Results

### Patient characteristics

A total of 129 patients were included, of whom 33 were diagnosed with IPA (4 proven; 29 probable) and bronchiectasis, 96 bronchiectasis patients without pulmonary aspergillosis served as controls. No significant differences were observed in terms of sex, age, BMI, Extra-pulmonary comorbidities, smoking history, history of glucocorticoid use and pulmonary function between the case and control groups (**Table 1**).

**Table 1:** Demographic characteristics of the study population.

Variables	IPA group (n = 33)	Control group (n = 96)	P value
Male	25	60	0.17
Age,y	64.09 ± 12.23	62.32 ± 11.60	0.46
BMI (Body Mass Index)	21.10 ± 3.89	21.90 ± 3.70	0.29
Extra-pulmonary comorbidities			
Cardiovascular diseases	12	37	0.82
Diabetes mellitus	4	8	0.50
*FEV1% predicted			
>=80%	10	30	0.60
50-79%	12	34	
30-49%	5	22	
<30%	6	10	
History of smoking	13	34	0.68
Steroid treatment	10	17	0.13

*FEV1: forced expiratory volume in 1 s.

### Galactomannan, Dectin-1 and IL-17 levels between IPA and non-IPA group

**Figure 1:**
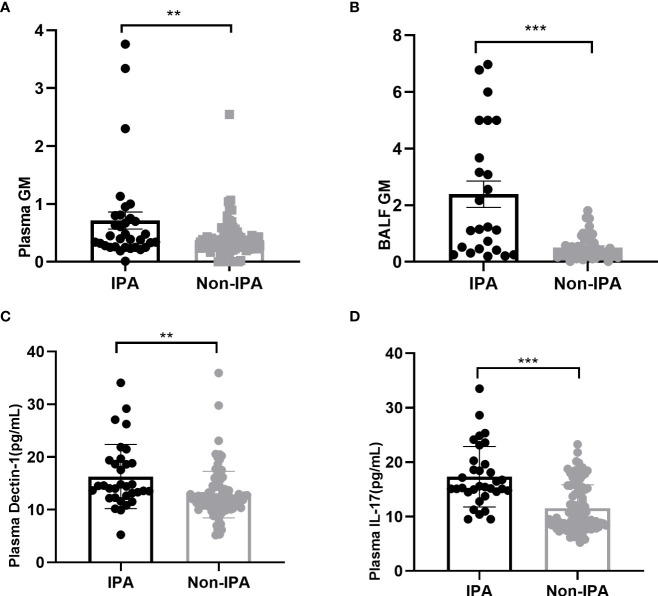
Plasma GM **(A)**, BALF GM **(B)**, plasma Dectin-1 **(C)** and IL-17 **(D)** were elevated in IPA group compared with the non-IPA group in patients with bronchiectasis. **P < 0.001; ***P < 0.001.

Our results revealed that plasma GM, and BALF GM levels were significantly elevated in IPA group than in non-IPA group (0.4[0.26-0.78]+ vs 0.31 [0.25-0.43], P=0.01; 1.19[0.43-4.67] vs 0.41[0.22-0.67], P<0.001, **Figure 1**).

The plasma Dectin-1 levels in the IPA group were significantly higher than that in the control group (16.29 ± 6.08 vs 12.87 ± 4.43pg/mL; P=0.005). Similarly, IL-17 levels in plasma were significantly elevated in the IPA group than in the non-IPA group (17.33 ± 5.55 vs11.56 ± 4.29pg/ml; P<0.001) (**Figure 1**).

### Diagnostic efficiency of Dectin-1, IL-17 and galactomannan

**Figure 2:**
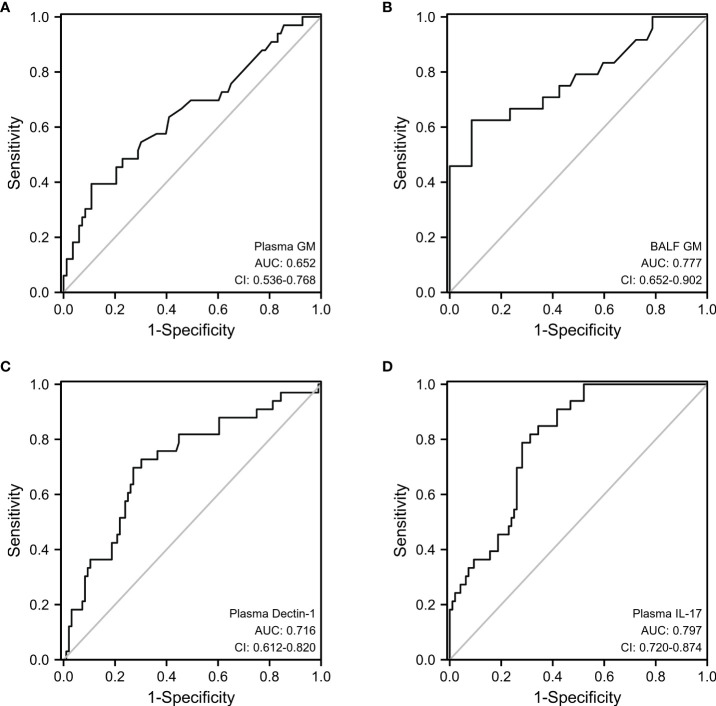
ROC analysis of plasma GM **(A)**, BALF GM **(B)**, plasma Dectin-1 **(C)**, and plasma IL-17 **(D)** to predict patients with bronchiectasis with IPA.

According to the ROC curve analysis, the best cutoff value for plasma GM for diagnosing IPA was 0.6, at which the sensitivity and specificity of the test were 39.4% and 89.2%, respectively (33 patients in the IPA group and 83 patients in the non-IPA group; AUC=0.652). When taking 1.01 as the cut-off value of GM, the sensitivity and specificity of GM in bronchoalveolar lavage fluid were 62.5% and 91.5%, respectively (24 patients in the IPA group and 47 patients in the non-IPA group; AUC=0.777; **Figure 2**).

We also performed a ROC curve analysis for Dectin-1 and IL-17, as shown in **Figure 2**. In optimal cutoff value for plasma Dectin-1 for diagnosing IPA was 13.29 ng/mL, at which the sensitivity and specificity were 69.7% and 72.9%, respectively (33 patients in the IPA group and 96 patients in the non-IPA group; AUC=0.716). Whereas, the sensitivity and specificity of IL-17 detection in serum were 78.8% and 71.9%, respectively (cut-off at 14.36pg/mL, 33 patients in the IPA group and 96 patients in the non-IPA group; AUC=0.797). The diagnostic sensitivity of plasma Dectin-1 and IL-17 was higher than that of plasma and BALF GM.

### Diagnostic accuracy of GM combined with IL-17 or Dectin-1 in IPA

**Figure 3:**
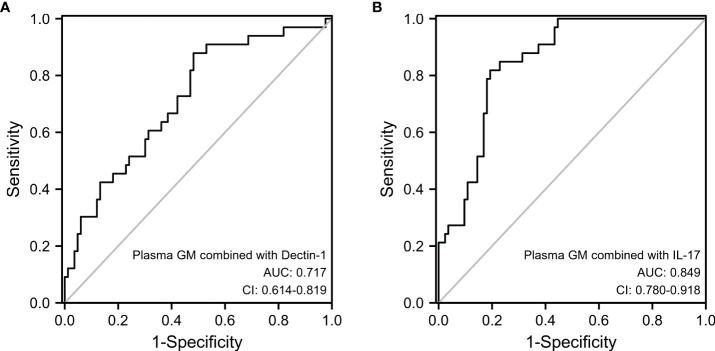
Diagnostic accuracy of combined testing of plasma GM and Dectin-1 **(A)** or plasma GM and IL-17 **(B)**.

The sensitivity and specificity of double positive plasma GM and dectin-1 in diagnosing IPA in patients with bronchiectasis were 87.9% and 51.8% (AUC=0.717). However, double positivity of the plasma GM and IL-17 tests for the diagnosis of IPA had a sensitivity of 81.8% and specificity of 80.7% (AUC=0.849). Compared with the plasma GM test, the combined diagnostic sensitivity of GM and IL-17 is significantly higher (p=0.0036), and there was no difference in specificity (p=0.19) (**Figure 3**).

### Correlation between plasma Dectin-1/IL-17 and various clinical indicators

We evaluated the association between plasma Dectin-1/IL-17 and various clinical indicators in patients with IPA and bronchiectasis. Our results showed that there was no significant linear correlation between Dectin-1/IL-17 and oxygenation index, PCT, CRP and albumin. However. plasma Dectin-1 and IL-17 showed positive linear correlations with bronchiectasis severity Index (BSI) score in linear regression. Moreover, plasma IL-17 was positively linear correlations with white cell count and neutrophil cell count (**Table 2**).

**Table 2:** Correlation between plasma Dectin-1/IL-17 and various clinical indicators in IPA combined with bronchiectasis group.

Clinical indicators	Dectin-1 (pg/mL)		IL-17 (pg/mL)	
	r	*P- value*	r	*P- value*
white cell count (×10^9/L)	0.275	0.122	0.418	**0.015**
neutrophil count (×10^9/L)	0.233	0.191	0.399	**0.021**
CRP(C-reactive protein)(mg/L)	0.110	0.542	0.165	0.358
PCT (procalcitonin)(ng/mL)	0.196	0.275	0.027	0.881
BSI score	0.455	**0.008**	0.579	**< 0.001**
Albumin (g/L)	0.176	0.328	-0.201	0.262
Oxygenation Index	-0.048	0.790	-0.170	0.345

Bold values means statistically significant.

## Discussion

Dectin-1 is a soluble type II transmembrane pattern recognition receptor that recognizes and binds to β-glucan of the *Aspergillus* cell wall. Further studies show that IL-17 production after *A.fumigatus* infection is dependent on Dectin-1. IL-17 is involved in neutrophil recruitment, activation and migration to sites of fungal infection. Several studies have shown that dectin-1 and IL-17 play critical roles in innate immunity to *Aspergillus* infection. In this study, The sensitivity of Dectin-1 and IL-17 for the diagnosis of IPA in patients with bronchiectasis was higher than that of GM. Combined use of GM and IL-17 detection can improve the diagnosis of IPA in bronchiectasis patients.

GM could be released into blood and BALF when *Aspergillus* invades human tissues. Therefore, the detection of GM may indicate *Aspergillus* infection. According to IDSA criteria, the GM test is considered to be the most specific biomarker for diagnosing IPA. Currently, few studies have reported on the diagnostic accuracy of GM testing for diagnosing IPA in patients with bronchiectasis. In this paper, according to the ROC curve analysis, the optimal plasma GM cutoff was 0.6, and the sensitivity and specificity were 39.4% and 89.2%, respectively. In studies of patients with non-neutropenic reported sensitivity of plasma GM has ranged from 11.6 to 90.9%, while specificity ranged from 66.3% to 100% (He et al., 2012; Zhang et al., 2013; Fortún et al., 2016). The sensitivity and specificity of the plasma GM test in our study of patients with bronchiectasis and IPA were within the range reported in previous studies. When the cutoff value was set at 1.01 in this study, the sensitivity of the BALF GM detection test was significantly higher than that of the plasma GM. Reports of ROC curve analysis in other studies showed that the optimal BALF GM cutoff values in non-neutropenic patients with pulmonary aspergillosis were between 0.7 and 1.25 (Zhou et al., 2017; He et al., 2021). Two other similar studies were conducted in China. The study by Zhang et al. showed that the optimal cutoff value for GM in BALF was 0.7 when diagnosing IPA in non-neutropenic patients, with a sensitivity of 72.97% (Zhou et al., 2017). The best cut-off value of another study was 0.87, with a sensitivity and specificity of 91.7% and 92.5%, respectively (Wu et al., 2021). The reason for the higher sensitivity than in the present study is that the patients in the above study included fewer patients with bronchiectasis(27.03%,29.2%).

Patients with bronchiectasis combined with IPA are prone to hemoptysis and severe respiratory failure, leading to intolerance of tracheoscopy and difficulty in obtaining alveolar lavage fluid. Studies have shown that *Aspergillus* infection could lead to elevated levels of several cytokines (Griffiths et al., 2021a; Yu et al., 2021). Therefore, it is important to find blood biomarkers for the diagnosis of IPA. At the cellular level, dectin-1 was mainly expressed in dendritic cells and mononuclear phagocytes, with lower expression in lymphocytes and eosinophils. In a study of human umbilical vein endothelial cells infected with *Aspergillus fumigatus*, it was found that dectin-1 was able to bind to the surface of *A. fumigatus* and dispersed inside and outside the cell. The expression of dectin-1 in cells infected with *Aspergillus* fumigation was significantly higher than that in the control group (Zhang et al., 2017). Previous *in vivo* studies have shown that mice without dectin-1 expression have significantly increased fungal load in lung tissue after nasal injection of *Aspergillus fumigatus* compared with wild-type mice (Werner et al., 2009). In Kalkanci et al ‘s study (Kalkanci et al., 2020), it was found that Dectin-1 expression was downregulated in invasive aspergillosis among the haematopoietic stem cell transplant group (0.1887 ± 0.072 vs 0.0655 ± 0.010), but not statistically significant. Similarly In a study of acute myeloid leukemia (AML) with Invasive fungal disease, it was found that Dectin-1 mRNA expression during hematopoietic regeneration was significantly lower compared to the later time point when the blood count was restored (Fischer et al., 2016). Previous studies have shown that AML patients who developed Invasive Aspergillosis were 10.2 times more likely to have low Dectin-1 expression (Griffiths et al., 2021b). These study shows that during hematologic disease induced immunosuppression, the body’s immune response to fungal pathogens declines due to decreased Dectin-1 mRNA levels. However, we explored that the expression of plasma Dectin-1 in IPA patients was significantly higher than that in controls. The reason for the opposite findings may be that the patients in the above study included immunocompromised patients whereas the present study included patients with relatively normal immune function. The ROC curve showed that the sensitivity of dectin-1 in diagnosing IPA was higher than that of GM, but the specificity was lower. The reason for the low specificity may be due to recent explorations suggesting that dectin-1 plays a wider role in defence against a variety of infectious diseases caused by pathogens including bacteria and viruses (Tone et al., 2019; Kalia et al., 2021).

The IL-17 family is a group of cytokines, mainly secreted by the CD4+ T helper cell subset, namely Th17 cells. IL-17 is a key inflammatory mediator in many diseases, such as autoimmunity, heart disease, and infection. In a study of rheumatoid arthritis (RA) (Qiu et al., 2022), it was found that IL17 combined with other cytokines can predict the incidence of osteoporosis complication in RA patients. Xu et al. (Xu et al., 2021) showed that the combination of IL-17 and IL-6 had the highest diagnostic accuracy in predicting the prognosis of left ventricular diastolic dysfunction by ROC curve. In the study of assessing the severity of coronary artery disease by Liu et al (Liu et al., 2022), it was also found that the clinical model containing IL17 had a good performance in predicting severe coronary artery disease. IL-17 plays a crucial role in defence against invasive infections caused by fungal pathogens of the *Aspergillus* by inducing antimicrobial proteins and recruiting neutrophils to the site of infection (Puerta-Arias et al., 2020). In a study of invasive pulmonary aspergillosis mice, it was found that IL-17A mRNA expression was significantly increased in the lung tissue in the IPA group (Xu et al., 2018). Kimura et al. found that IL-17 levels in bronchoalveolar lavage fluid of mice infected with *Aspergillus fumigatus* increased gradually as GM levels, and posaconazole treatment significantly reduced IL-17 level (Kimura et al., 2017). In the COPD combined IPA mouse model, the serum IL-17 level in the COPD+IPA group was significantly higher than that in the COPD group (17.96 ± 9.59 pg/mL vs. 8.05 ± 4.44 pg/mL, P = 0.02) (Geng et al., 2020). In two studies of fungal sinusitis (Rai et al., 2018b; Rai et al., 2018a), IL-17 serum levels were significantly higher in these patients than in controls. The above results are similar to our study. However, Delsing et al’s study found that *Aspergillus*-specific Th17 responses (production of IL-17) were significantly reduced in patients with *Aspergillus* skull base osteomyelitis compared to the healthy control group (Delsing et al., 2015). The reason for the different results is that the study by Delsing et al. included only 6 patients, one of whom had chronic myeloid leukemia (immunosuppressed population). The study by Hassan et al. showed that in fungal infections in patients with end-stage liver disease, patients with invasive fungal infection (41.67% of the patients had *Aspergillus* infection)had higher serum IL-17 levels compared with patients without invasive fungal infection (Hassan et al., 2014). Our research is consistent with the above findings. We found IL-17 levels in plasma were significantly elevated in the IPA group than in the non-IPA group. At the optimal cutoff value, The sensitivity of plasma IL-17 is significantly higher than that of plasma and BALF GM, but the specificity is lower than that of plasma and BALF GM.

When plasma GM combined with dectin-1 was used to diagnose IPA, although the sensitivity was higher (87.9%), the specificity was significantly lower (51.8%), which may lead to misdiagnosis. However, the diagnostic sensitivity (81.8%) of plasma GM combined with IL-17 in IPA was significantly higher than that of plasma GM, while the specificity (80.7%) was not significantly different. These findings suggest that plasma GM combined with IL-17 can increase the sensitivity of the diagnosis of IPA in patients with bronchiectasis.

In our study, plasma IL-17 was positively linear correlations with white cell count and neutrophil cell count. The reason may be related to the recruitment and activation of neutrophils by IL-17, which can effectively promote airway inflammation (Ritzmann et al., 2022). The bronchiectasis severity index (BSI) was used to evaluate the severity of bronchiectasis. In our study, plasma Dectin-1 and IL-17 showed positive linear correlations with BSI score in linear regression. These results suggest that Dectin-1 and IL-17 may reflect the severity of the disease in patients with pulmonary aspergillosis. However, this needs to be validated in larger numbers of patients.

One of the weaknesses of the current study is the lack of dectin-1and IL-17 detection in bronchoalveolar lavage fluid which we aim to address in future work. Furthermore, future studies need to be validated in a larger cohort and include more non-neutropenic patients.

## Conclusions

In conclusion, the sensitivity of Dectin-1 and IL-17 was better than that of GM for the diagnosis of IPA in patients with bronchiectasis. The combination of GM and IL-17 in plasma is helpful for the diagnosis of IPA in bronchiectasis, especially in patients who cannot perform invasive procedures.

## Data availability statement

The raw data supporting the conclusions of this article will be made available by the authors, without undue reservation.

## Ethics statement

The studies involving human participants were reviewed and approved by the Institutional Review Board of Changzhou first people’s Hospital. The patients/participants provided their written informed consent to participate in this study.

## Author contributions

QH and CF designed the study. JC, ML, and MZ collected and analyzed the data. QH and CF wrote the paper. All authors contributed to the article and approved the submitted version.

## Funding

This work was supported by the Changzhou Young Talent Technology Project (grant number: QN201802).

## Conflict of interest

The authors declare that the research was conducted in the absence of any commercial or financial relationships that could be construed as a potential conflict of interest.

## Publisher’s note

All claims expressed in this article are solely those of the authors and do not necessarily represent those of their affiliated organizations, or those of the publisher, the editors and the reviewers. Any product that may be evaluated in this article, or claim that may be made by its manufacturer, is not guaranteed or endorsed by the publisher.

## References

[B1] Dai Z. Cai M. Yao Y. Zhu J. Lin L. Fang L.. (2021). Comparing the diagnostic value of bronchoalveolar lavage fluid galactomannan, serum galactomannanan, and serum 1,3-β-d-glucan in non-neutropenic respiratory disease patients with invasive pulmonary aspergillosis. Med. (Baltimore) 100, e25233. doi: 10.1097/MD.0000000000025233 PMC8036023 33832082

[B2] Delsing C. E. Becker K. L. Simon A. Kullberg B. J. Bleeker-Rovers C. P. van de Veerdonk F. L.. (2015). Th17 cytokine deficiency in patients with aspergillus skull base osteomyelitis. BMC Infect. Dis. 15, 140. doi: 10.1186/s12879-015-0891-2 25888308 PMC4374583

[B3] Donnelly J. P. Chen S. C. Kauffman C. A. Steinbach W. J. Baddley J. W. Verweij P. E.. (2020). Revision and update of the consensus definitions of invasive fungal disease from the European organization for research and treatment of cancer and the mycoses study group education and research consortium. Clin. Infect. Dis. 71, 1367–1376. doi: 10.1093/cid/ciz1008 31802125 PMC7486838

[B4] Fischer M. Spies-Weisshart B. Schrenk K. Gruhn B. Wittig S. Glaser A.. (2016). Polymorphisms of dectin-1 and TLR2 predispose to invasive fungal disease in patients with acute myeloid leukemia. PloS One 11, e0150632. doi: 10.1371/journal.pone.0150632 26963509 PMC4786091

[B5] Fortún J. Martín-Dávila P. Gomez Garcia de la Pedrosa E. Silva J. T. Garcia-Rodríguez J. Benito D.. (2016). Galactomannan in bronchoalveolar lavage fluid for diagnosis of invasive aspergillosis in non-hematological patients. J. Infect. 72, 738–744. doi: 10.1016/j.jinf.2016.02.019 27025205

[B6] Geng W. R. He H. Y. Zhang Q. Tong Z. H. (2020). Th17 cells are involved in mouse chronic obstructive pulmonary disease complicated with invasive pulmonary aspergillosis. Chin. Med. J. 134, 555–563. doi: 10.1097/CM9.0000000000001183 33323817 PMC7929714

[B7] Gold J. Revis A. Thomas S. Perry L. Blakney R. A. Chambers T.. (2022). Clinical characteristics, health care utilization, and outcomes among patients in a pilot surveillance system for invasive mold disease-Georgia, united states 2017-2019. Open Forum Infect. Dis. 9, ofac215. doi: 10.1093/ofid/ofac215 35794945 PMC9253885

[B8] Griffiths J. S. Camilli G. Kotowicz N. K. Ho J. Richardson J. P. Naglik J. R. (2021a). Role for IL-1 family cytokines in fungal infections. Front. Microbiol. 12. doi: 10.3389/fmicb.2021.633047 PMC7902786 33643264

[B9] Griffiths J. S. White P. L. Thompson A. da Fonseca D. M. Pickering R. J. Ingram W.. (2021b). A novel strategy to identify haematology patients at high risk of developing aspergillosis. Front. Immunol. 12. doi: 10.3389/fimmu.2021.780160 PMC8716727 34975870

[B10] Hassan E. A. Abd El-Rehim A. S. Hassany S. M. Ahmed A. O. Elsherbiny N. M. Mohammed M. H. (2014). Fungal infection in patients with end-stage liver disease: low frequency or low index of suspicion. Int. J. Infect. Dis. 23, 69–74. doi: 10.1016/j.ijid.2013.12.014 24726663

[B11] He H. Ding L. Sun B. Li F. Zhan Q. (2012). Role of galactomannan determinations in bronchoalveolar lavage fluid samples from critically ill patients with chronic obstructive pulmonary disease for the diagnosis of invasive pulmonary aspergillosis: a prospective study. Crit. Care 16, R138. doi: 10.1186/cc11443 22835335 PMC3580723

[B12] He Q. Zhang M. Feng C. (2021). The role of pentraxin3 in plasma and bronchoalveolar lavage fluid in COPD patients with invasive pulmonary aspergillosis. BMC Pulm Med. 21, 414. doi: 10.1186/s12890-021-01793-z 34915889 PMC8680116

[B13] Kalia N. Singh J. Kaur M. (2021). The role of dectin-1 in health and disease. Immunobiology 226, 152071. doi: 10.1016/j.imbio.2021.152071 33588306

[B14] Kalkanci A. Tug E. Fidan I. Guzel Tunccan O. Ozkurt Z. N. Yegin Z. A.. (2020). Retrospective analysis of the association of the expression and single nucleotide polymorphisms (SNPs) of the TLR4, PTX3 and dectin-1 (CLEC/A) genes with development of invasive aspergillosis among haematopoietic stem cell transplant recipients with oncohaematological disorders. Mycoses 63, 832–839. doi: 10.1111/myc.13087 32291814

[B15] Kimura G. Nakaoki T. Nishimoto Y. Suzuki Y. Rapeport G. Strong P.. (2017). Effects of intranasally dosed posaconazole on fungal load and biomarkers in aspergillus fumigatus infected immunocompromised mice. Mycoses 60, 728–735. doi: 10.1111/myc.12653 28699245

[B16] Liu S. Wang C. Guo J. Yang Y. Huang M. Li L.. (2022). Serum cytokines predict the severity of coronary artery disease without acute myocardial infarction. Front. Cardiovasc. Med. 9. doi: 10.3389/fcvm.2022.896810 PMC9149173 35651907

[B17] Mac Aogáin M. Chandrasekaran R. Lim A. Low T. B. Tan G. L. Hassan T.. (2018). Immunological corollary of the pulmonary mycobiome in bronchiectasis: the CAMEB study. Eur. Respir. J. 52(1), 1800766. doi: 10.1183/13993003.00766-2018 29880655 PMC6092680

[B18] Patterson T. F. Thompson G. R. 3rd Denning D. W. Fishman J. A. Hadley S. Herbrecht R.. (2016). Practice guidelines for the diagnosis and management of aspergillosis: 2016 update by the infectious diseases society of America. Clin. Infect. Dis. 63, e1–1e60. doi: 10.1093/cid/ciw326 27365388 PMC4967602

[B19] Puerta-Arias J. D. Mejía S. P. González Á. (2020). The role of the interleukin-17 axis and neutrophils in the pathogenesis of endemic and systemic mycoses. Front. Cell Infect. Microbiol. 10. doi: 10.3389/fcimb.2020.595301 PMC7793882 33425780

[B20] Qiu J. Lu C. Zhang L. Zhou X. Zou H. (2022). Osteoporosis in patients with rheumatoid arthritis is associated with serum immune regulatory cellular factors. Clin. Rheumatol. 41(9), 2685–2693. doi: 10.1007/s10067-022-06212-0 35670881

[B21] Rai G. Ansari M. A. Dar S. A. Datt S. Gupta N. Sharma S.. (2018b). Serum cytokine profile in patients with chronic rhinosinusitis with nasal polyposis infected by aspergillus flavus. Ann. Lab. Med. 38, 125–131. doi: 10.3343/alm.2018.38.2.125 29214756 PMC5736671

[B22] Rai G. Das S. Ansari M. A. Singh P. K. Gupta N. Sharma S.. (2018a). Phenotypic and functional profile of Th17 and treg cells in allergic fungal sinusitis. Int. Immunopharmacol 57, 55–61. doi: 10.1016/j.intimp.2018.02.009 29475096

[B23] Ritzmann F. Lunding L. P. Bals R. Wegmann M. Beisswenger C. (2022). IL-17 cytokines and chronic lung diseases. Cells 11(14):2132. doi: 10.3390/cells11142132 35883573 PMC9318387

[B24] Sullivan A. Hunt E. B. Ward C. Lapthorne S. Eustace J. A. Fanning L. J.. (2020). The presence of aspergillus fumigatus in asthmatic airways is not clearly related to clinical disease severity. Allergy 75, 1146–1154. doi: 10.1111/all.14087 31605638

[B25] Tiew P. Y. Mac Aogáin M. Ter S. K. Aliberti S. Chalmers J. D. Chotirmall S. H. (2021). Respiratory mycoses in COPD and bronchiectasis. Mycopathologia 186, 623–638. doi: 10.1007/s11046-021-00539-z 33709335

[B26] Tone K. Stappers M. Willment J. A. Brown G. D. (2019). C-type lectin receptors of the dectin-1 cluster: Physiological roles and involvement in disease. Eur. J. Immunol. 49, 2127–2133. doi: 10.1002/eji.201847536 31580478 PMC6916577

[B27] Werner J. L. Metz A. E. Horn D. Schoeb T. R. Hewitt M. M. Schwiebert L. M.. (2009). Requisite role for the dectin-1 beta-glucan receptor in pulmonary defense against aspergillus fumigatus. J. Immunol. 182, 4938–4946. doi: 10.4049/jimmunol.0804250 19342673 PMC3434356

[B28] Wu Z. Wang L. Tan L. Wu J. Chen Z. Hu M. (2021). Diagnostic value of galactomannan in serum and bronchoalveolar lavage fluid for invasive pulmonary aspergillosis in non-neutropenic patients. Diagn Microbiol. Infect. Dis. 99, 115274. doi: 10.1016/j.diagmicrobio.2020.115274 33453546

[B29] Xu J. F. Gao Y. H. Song Y. L. Qu J. M. Guan W. J. (2022). Research advances and clinical management of bronchiectasis: Chinese perspective. ERJ Open Res. 8, 00017-2022. doi: 10.1183/23120541.00017-2022 35415184 PMC8995535

[B31] Xu L. N. Xu R. A. Zhang D. Su S. S. Xu H. Y. Wu Q.. (2018). The changes of expressive levels of IL-17A, STAT3, and RORγt in different invasive pulmonary aspergillosis mice. Infect. Drug Resist. 11, 1321–1328. doi: 10.2147/IDR.S172949 30214252 PMC6118236

[B30] Xu L. Yan J. Zhang F. Zhou C. Fan T. Chen X.. (2021). Use of inflammatory biomarkers and real-time cardiac catheterisation to evaluate the left ventricular diastolic function in patients with diastolic heart failure. Heart Lung Circ. 30, 396–403. doi: 10.1016/j.hlc.2020.06.017 32736962

[B32] Yang B. Kim T. Ryu J. Park H. Y. Hwangbo B. Kong S. Y.. (2021). Increased incidence and associated risk factors of aspergillosis in patients with bronchiectasis. J. Pers. Med. 11(5), 422. doi: 10.3390/jpm11050422 34067607 PMC8155934

[B33] Yu M. Song X. T. Liu B. Luan T. T. Liao S. L. Zhao Z. T. (2021). The emerging role of mast cells in response to fungal infection. Front. Immunol. 12. doi: 10.3389/fimmu.2021.688659 PMC8209461 34149729

[B35] Zhang X. B. Chen G. P. Lin Q. C. Lin X. Zhang H. Y. Wang J. H. (2013). Bronchoalveolar lavage fluid galactomannan detection for diagnosis of invasive pulmonary aspergillosis in chronic obstructive pulmonary disease. Med. Mycol. 51, 688–695. doi: 10.3109/13693786.2013.777162 23527739

[B34] Zhang P. P. Xin X. F. Xu X. Y. Fang L. P. Wu J. Shi Y. (2017). Toll-like receptor 2 and dectin-1 function as promising biomarker for aspergillus fumigatus infection. Exp. Ther. Med. 14, 3836–3840. doi: 10.3892/etm.2017.5000 29042988 PMC5639428

[B37] Zhou W. Li H. Zhang Y. Huang M. He Q. Li P.. (2017). Diagnostic value of galactomannan antigen test in serum and bronchoalveolar lavage fluid samples from patients with nonneutropenic invasive pulmonary aspergillosis. J. Clin. Microbiol. 55, 2153–2161. doi: 10.1128/JCM.00345-17 28446576 PMC5483917

[B36] Zhou P. Xie Y. Yan Z. Liu X. Hua H. (2019). Association between dectin-1 gene single nucleotide polymorphisms and fungal infection: a systemic review and meta-analysis. Biosci. Rep. 39(11):BSR20191519. doi: 10.1042/BSR20191519 31696220 PMC6851518

